# Representations of Value in the Brain: An Embarrassment of Riches?

**DOI:** 10.1371/journal.pbio.1002174

**Published:** 2015-06-18

**Authors:** Jeffrey J. Stott, A. David Redish

**Affiliations:** 1 Graduate Program in Neuroscience, University of Minnesota, Minneapolis, Minnesota, United States of America; 2 Department of Neuroscience, University of Minnesota, Minneapolis, Minnesota, United States of America

## Abstract

Over the past two decades, neuroscientists have increasingly turned their attention to the question of how the brain implements decisions between differently valued options. This emerging field, called neuroeconomics, has made quick progress in identifying a plethora of brain areas that track or are modulated by reward value. However, it is still unclear how and where in the brain value coding takes place. A primate study by Strait and colleagues in this issue of *PLOS Biology* finds overlapping signals of value coding in two brain regions central to the valuation process: the ventromedial prefrontal cortex and the ventral striatum. This finding reconciles the primate and rodent literatures, provides valuable insight into the complexity of value computation, and helps set the agenda for future work in this area.

## Abstract 2

This Primer examines a study that finds remarkably similar value-related information in two distinctly different brain regions, thereby begging the question, “do we really understand how the brain calculates value?” Read the Research Article.

## The (Neuro)Economic Model of Choice

A central axiom of economic models of choice is that there exists a single, common scale of value that the decisionmaker uses to compare options, and that this common currency can be revealed through the actions of the animal (see Box 1 for a glossary of terms used in this preview). This hypothesis matches our intuitive notion that when we make decisions, we weigh them against each other. Mathematically, this principle is necessary for internally consistent (i.e., rational) choices. Decisions arise from computations occurring in neural systems. Under the assumption of this central axiom of economic choice, one would expect to find abstract representations of value somewhere in the nervous system. Therefore, it generated a great deal of excitement when neuroscientists studying decision making started finding correlates of value within the brain [1].

**Box 1. Glossary of Terms**

Value: An integration of factors that affect how desirable a reward is, including the magnitude of reward, probability of receiving the reward, delay to the reward, effort needed to obtain the reward, etc. Economics tells us that value has to be revealed through an agent’s actions.

Subjective value: Because value reflects the specific needs and preferences of the individual, value is inherently subjective. Subjective value is contrasted with objective value.

Common currency: A metric for measuring or representing the value of an option so that all things can be measured on the same scale. In neural terms, a common currency coding scheme refers to a reliable relationship between the activity of a neuron (or brain region) and the value of a specific option.

State-based representations: Representations about the “state of the world.” In terms of reward, a state-based representation could include factors beyond immediate value such as reward identity (i.e., flavor), when or where the reward occurs (contextual information), the motor action needed to obtain the reward (i.e., “go left” versus “go right”), etc.

Expectations of reward: Explicit knowledge about potential rewards. In neural terms, this would be reflected in changes in neural firing that are elicited by cues (external or internally generated) that predict reward.

Orbitofrontal cortex (OFC): Part of the prefrontal cortex located behind the orbit of the eyes (in primates) and in the anterior (frontal) region of the cerebral cortex in rodents. The OFC is activated by reward receipt and by stimuli that predict reward.

Ventromedial prefrontal cortex (vmPFC): Part of the prefrontal cortex medial to the OFC. However, some papers use these two terms synonymously. The vmPFC is activated by reward receipt and by stimuli that predict reward.

Ventral striatum (VS): The striatum is the major input nucleus of the basal ganglia, a series of interconnected brain nuclei that form a loop with the cerebral cortex. The ventral (toward the bottom) aspect of the striatum is activated by rewards and reward-predicting stimuli. It also receives dense inputs from the dopamine system, and this input is thought to mediate reward learning.

A number of studies that recorded activity from single neurons in the monkey orbitofrontal cortex (OFC) found neurons that changed their firing rate according to the subjective value of the reward being offered, showing a common currency for value, independent of (or “abstracted from”) the sensory and motor variables of the task [2]. In agreement with this model, previous experiments by Strait and colleagues have found evidence for common currency signaling in a nearby cortical structure, the ventromedial prefrontal cortex (vmPFC) [3].

In contrast, neuroeconomic experiments in rodents have largely failed to find these representations of abstract value signaling in the orbitofrontal cortex, instead finding more state-based representations of expectations of reward [4–9]. Moreover, the rodent studies have identified a prominent role for value coding in the ventral striatum (VS) [9–13], calling into question a unique role for the OFC in signaling value.

While it is certainly possible that this discrepancy could be chalked up to species-specific differences in brain architecture [14], and/or differences in task design, the study reported in this issue of *PLOS Biology* by Strait and colleagues [15] argues against these interpretations. Strait et al. find overlapping signatures of value coding in monkey vmPFC and VS, including evidence for abstract value coding, as required by the common currency model of decision making.

This result highlights a fundamental open question in the field of neuroeconomics: If multiple brain regions contain value signals, are these brain regions performing similar or distinct functions during the decision-making process? How do we account for the multiplicity of value signals found throughout the brain?

## Computation

The fundamental question to ask about neural signals is: what computation are these signals providing to the behavior? Neural systems are computational processes evolved to select appropriate actions from information taken from one’s past (memory), one’s needs and goals (motivation), and one’s information about the world (sensory cues) [16].

### The Question of Timing

Comparing the timecourse of representations in the vmPFC and VS, Strait et al. found that neural signals related to the value of the chosen action emerged earlier in the VS [15]. Interestingly, neural activity in the VS was able to predict the animal’s decision ahead of the choice period, but the neural activity in the vmPFC did not.

**Fig 1:**
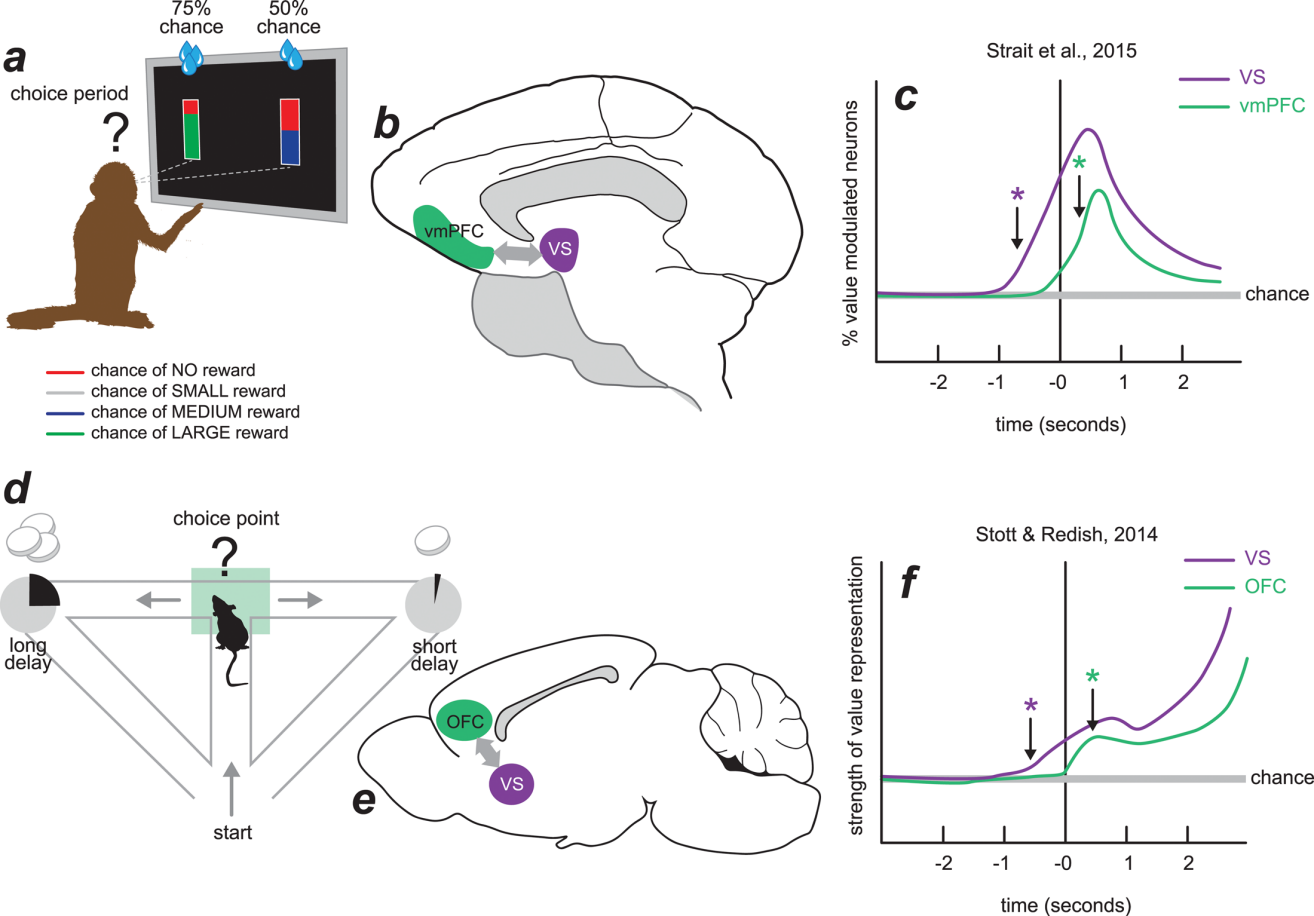
Schematic illustration of parallel neuroeconomic tasks in monkey and rat. The top panel illustrates the task (A), recording locations (B), and one of the significant results (C) from the study highlighted in this issue of *PLOS Biology* by Strait and colleagues[15]. The bottom panel illustrates the task (D), recording locations (E), and one of the significant results (F) from the study by Stott and Redish, 2014[9]. *Image credit: Karin Odell.*

These results closely match a recent study in rodents where neural signals were recorded simultaneously from the OFC and VS in rats performing a neuroeconomic decision-making task [9]. Fig 1 shows a side-by-side comparison of the recording study in monkeys by Strait et al. [15] featured in this issue with a recording study in rodents by Stott and Redish [9]. In the study by Stott and Redish, reward expectation coding occurred earlier in the VS than in the OFC during trials in which the rat deliberated at the choice point (Fig 1D–1F). Strait et al. find a similar result of an earlier appearance of value-related signals in the VS than the vmPFC in the monkey (Fig 1A–1C). Both studies show that the VS contains value-related information before the OFC/vmPFC, hinting at potentially different roles for these structures, despite their similarity along other dimensions of value signaling [9,15]. In particular, the precise timing of activity in these two brain regions suggests that the VS activity arises earlier in the chain of events leading to action selection than the OFC or vmPFC.

## A Multiplicity of Value Signals in the Brain

Neuroeconomic experiments have identified a number of brain regions that exhibit neural responses related to reward value [9,17–22]. The data presented by Strait et al. [15] in this issue of *PLOS Biology* adds to this body of work, showing similar representations of value in the VS and vmPFC on the same task, albeit with differences in the timing of activity. Still, the broad overlap in value signaling between different structures invites the question of what these multiple brain regions are contributing to choice. There are three hypotheses that could explain the presence of value-related neural firing in multiple brain structures.

First, trivially, multiple structures could be contributing the same valuation computations to the system, at which point one would expect to see multiple copies of value-related signals. This hypothesis is unlikely to be correct, because studies have consistently found subtle differences between the representations of value, and because value-related signals have been found across the brain, including in structures well-known to be involved in other functions (such as sensorimotor integration [17]) and even in primary sensory areas such as the visual cortex [23].

Second, value representations could serve as a modulator of other signals. Value is important in a number of domains, including sensory, motor, and cognitive activities. In perceptual discrimination tasks, increasing the value of a target has been shown to increase neural firing in cells that detect sensory stimuli [23], in cells that are tuned to specific motor actions [24], and in cells that are more aptly described as “sensorimotor,” integrating sensory and motor information [17]. Therefore, value signals are important in focusing attention toward salient stimuli and in biasing motor commands toward more valuable actions [25]. These forms of secondary modulation (wherein a cell has a primary response to a stimulus or concept but shows firing rate modulation of that primary tuning) occur throughout the brain, such as attentional modulation of visual stimuli in the primary visual cortex [23] or task-related modulation of place representations in the hippocampus [26].

Third, when interpreting the role of different brain regions involved in choice, it is important to keep in mind that the brain may accomplish a given task using a variety of different solutions. A broad body of work now supports the view that the brain contains several functionally and anatomically distinct decision-making systems, each of which selects actions (makes decisions) using a different underlying computational algorithm [16,27,28]. These different decision-making systems could also be considered as different valuation systems [16,29], under the assumption that decisions reflect value. This interpretation could explain the existence of overlapping value signals seen in the vmPFC and VS. Both areas may routinely generate representations of value, as seen by Strait et al., but these brain areas may be differentially recruited depending on which action selection system is being engaged.

Each of these hypotheses call into question the centralized common currency hypothesis of value and opens new unanswered questions.

## The *Ignorance* Questions

In his intriguing book *Ignorance* [30], Stuart Firestein suggests that the purpose of science is less to identify new answers as much as it is to identify new questions: what are the questions that you didn’t know were questions until you made this discovery? These new observations that value representations occur across the brain open up several new questions and call into question even the fundamental concept of value in a common currency.

### What Happens When These Valuations Come into Conflict?

If there are multiple representations of value, then there must either be a mechanism to ensure that they always retain the same ordering between options or there must be a process by which the agent can take an action in the face of conflicting value signals. There is an extensive literature in behavioral economics showing irrational behavior (i.e., when choices are influenced by aspects that should not logically factor into the decision) under specific situations. Examples include framing effects, loss aversion, the endowment effect, extremeness aversion, sunk costs, etc. [31,32]. Do these irrationalities arise from conflict between multiple representations of value?

### Do These Systems Participate in a Single Valuation Circuit?

One possible explanation for multiple valuation representations in the brain is that they are part of a larger circuit interacting with each other. This, of course, raises the question of the anatomical and functional connectivity between these neural structures. There is now extensive evidence that functional circuits can change on a moment to moment basis within tasks [33,34]. Is the functional flow of information from the VS to the OFC and vmPFC, as suggested by the timing data [9,15]? Or does it depend on the specifics of the task involved? In rats, lesion studies suggest that the VS and OFC are necessary for different aspects of valuation, depending on different task parameters, with the OFC only playing a role when flavor and identity are involved (apples versus oranges), but the VS playing a more general role whenever value is compared (both one apple to three apples and apples versus oranges) [4].

### What Are the Computational Differences between These Different Valuation Signals?

The idea that there are multiple decision-making systems that can calculate value in different ways [16, 27, 29] predicts the presence of many different value signals in the brain. Although any given action may be driven by one of these systems, neural systems are often active even in tasks in which they are not playing an immediate computational role. For example, hippocampal neurons show place fields on tasks that do not require an intact hippocampus [26].

## The Concept of Value

As intriguing and logical as the common currency concept is, it has recently come under scrutiny [35]. Strait et al. [15] and others [1] have found multiple representations of value in the brain, but we do not yet know if these representations must agree with each other or not. If they do not, then we come to an interesting question of arbitration and mediation and what drives behavior in these conflict situations. The relationship between these valuation signals and the decisions being made by the animal is a very intriguing open and interesting question that will require additional experiments.

## Abbreviations

OFC: orbitofrontal cortex
vmPFC: ventromedial prefrontal cortex
VS: ventral striatum
